# AGING WELL, AGING DIFFERENTLY: A COMPARISON BETWEEN LIFE PLAN COMMUNITY RESIDENTS AND THE COMMUNITY AT LARGE

**DOI:** 10.1093/geroni/igad104.2004

**Published:** 2023-12-21

**Authors:** Laurel Mertz, Jennifer Smith, Nicole Lehpamer, Ajla Basic, Catherine O’Brien

**Affiliations:** Mather, Evanston, Illinois, United States; Mather, Evanston, Illinois, United States; Mather Institute, Evanston, Illinois, United States; Mather, Evanston, Illinois, United States; Mather, Evanston, Illinois, United States

## Abstract

Residing in Life Plan Communities (or Continuing Care Retirement Communities) may have benefits to well-being due to greater access to wellness programs and services. The purpose of this research was to examine changes in health and wellness between Life Plan Community residents and older adults residing in the community at large over five years. Data included 1,729 residents (69% female, mean baseline age = 81.64) who participated in Years 1 and 5 of the Age Well Study, and 427 older adults (50% female, mean baseline age = 80.78) residing in the community at large who participated in multiple waves of the Health and Retirement Study. The community-at-large group was proportionally sampled to be similar to the residents on age, income, and race/ethnicity, and both groups completed the same measures of health and wellness at each time point. Data were analyzed using multilevel models controlling for age, gender, income, education, and marital status. Compared to the community-at-large older adults, residents tended to exhibit better physical, emotional, social, intellectual, and vocational wellness in Years 1 and 5, but lower spiritual wellness in Year 5. When comparing Year 1 and Year 5, both groups reported similar changes over time on physical and vocational wellness measures. Community-at-large older adults tended to report more favorable changes in emotional and spiritual wellness, and residents showed more favorable changes in social and intellectual wellness. Although effects of the pandemic should be explored further, these findings have implications for supporting aging well in different types of residential settings.

